# Supplementary material for: Alterations in Microbiota and Metabolites Related to Spontaneous Diabetes and Pre-Diabetes in Rhesus Macaques
Source: Genes (Basel). 2022 Aug 24;13(9):1513. doi: 10.3390/genes13091513 (PMC9498908; doi:10.3390/genes13091513)
Supplement: Supplementary file 1 [file genes-13-01513-s001.zip › supplementary figure legends.pdf]

Figure legends

Supplementary Figure S1 The Shannon index, Observrd-otus index and Evenness index between T2DM, IGR and Control groups (A). Principal Co-ordinates Analysisanalysis (PCoA) of beta diversity was based on the Jaccard and unweighted-UniFrac between the T2DM and Control groups (B). Principal Co-ordinates Analysisanalysis (PCoA) of beta diversity was based on the Jaccard and unweighted-UniFrac between the IGR and Control groups (C).

Supplementary Figure S2 The microbial composition between T2DM, IGR and Control groups at the phyla level (A), the family level (B) and the genus level(C).

Supplementary Figure S3 Functional differences of gut microbes between T2DM and Control groups (A), IGR and Control groups (B) ($P<0.1$). Orange box: T2DM or IGR samples, blue box: controls.

Supplementary Figure S4 Orthogonal projection to latent structures discrimination analysis (OPLS-DA) score plots between T2DM and Control groups (A), IGR and Control groups (B) in negtive model.
