# Supplementary material for: Alterations in Microbiota and Metabolites Related to Spontaneous Diabetes and Pre-Diabetes in Rhesus Macaques
Source: Genes (Basel). 2022 Aug 24;13(9):1513. doi: 10.3390/genes13091513 (PMC9498908; doi:10.3390/genes13091513)
Supplement: Supplementary file 1 [file genes-13-01513-s001.zip › Supplementary Tables S1-S5.pdf]

Supplementary Table S1. Basic information of T2DM，IGR and healthy subjects

ID	Group	Gender	BMI	Age	FPG （mmol/L）	Hb1AC （%）	FPI （μU/mL）	IR	TG （mmol/L）	TC （mmol/L）	HDL （mmol/L）	LDL （mmol/L）
MA-12	T2DM	M	16.8	8	9.88	4.0	18.09	7.94	0.47	3.79	1.31	1.68
MA-13	T2DM	M	15	7	9.37	2.9	10.70	4.46	0.45	3.45	1.57	1.13
MA-17	T2DM	M	16.7	8	7.78	3.6	20.31	7.02	0.40	3.04	1.40	1.04
MA-18	T2DM	M	16.7	7	7.13	4.1	13.15	4.17	0.43	3.58	1.49	1.54
MA-29	T2DM	F	15	19	15.79	10.9	4.61	3.24	2.49	3.10	1.07	0.88
MA-6	T2DM	F	13.1	17	21.61	4.6	12.36	11.87	0.45	2.73	1.04	1.22
MA-9	T2DM	F	20	22	7.61	4.6	34.75	11.75	1.56	4.51	1.64	1.95
MA-11	IGR	M	13.1	6	6.88	3.7	10.62	3.25	0.79	2.49	0.72	1.16
MA-15	IGR	M	14	10	6.41	4.3	12.30	3.50	0.57	4.71	1.73	2.01
MA-16	IGR	M	15.3	12	6.21	3.4	12.38	3.42	1.01	2.72	0.99	1.02
MA-20	IGR	M	13.6	7	6.67	4.7	13.38	3.97	0.40	3.44	1.27	1.49
MA-30	IGR	F	15	10	6.31	4.1	10.67	2.99	0.76	4.89	1.97	2.17
MA-1	Control	M	16.6	7	2.63	3.6	5.73	0.67	0.54	2.37	0.84	0.94
MA-5	Control	F	14.8	12	3.35	2.3	6.05	0.90	0.37	1.98	0.71	0.51
MA-7	Control	F	13.5	11	4.04	3.8	2.86	0.51	0.41	4.13	1.80	1.63
MA-19	Control	M	14.0	8	4.67	3.5	9.73	2.02	0.37	2.50	1.25	0.84
MA-26	Control	M	17.9	7	4.18	3.1	5.49	1.02	0.40	4.05	1.77	1.66

MA-28	Control	M	15.2	7	5.02	3.4	4.27	0.95	0.54	3.39	1.41	1.44
MA-2	Control	F	15.3	18	2.99	2.4	7.95	1.06	0.52	4.54	1.72	2.06
MA-3	Control	M	14.2	18	5.49	3.6	8.28	2.02	0.47	3.76	1.29	1.89
MA-22	Control	M	14.0	7	3.43	3.4	2.61	0.40	0.57	3.67	1.18	1.99
MA-23	Control	M	15	7	5.21	4.1	6.77	1.57	0.51	3.05	1.25	1.10

Supplementary Table S2. Fecal metabolites with differential abundances between T2DM and Control groups

Adduct	Description	VIP	Fold change	*p*-value
（M+H）+	.beta.-Homoproline	1.381417321	2.183921481	0.03206583
（M+CH3COO+2H）+	cis-9-Palmitoleic acid	1.093888036	1.714189497	0.020383255
（M+H）+	Indole-2-carboxylic acid	21.66206774	4.579627587	0.009937014
（M+H）+	Leu-Arg	2.453313917	0.450591025	0.040654234
（M+CH3CN+H）+	L-Proline	1.302438103	2.040436553	0.015518049
（M+H）+	N2-Acetyl-L-ornithine	1.912103984	1.897632013	0.038607804
（M+H）+	Oxyquinoline	8.26639192	2.186529519	0.024133631
（M+CH3CN+H）+	Pyridoxine	1.35169432	0.635341046	0.022487808
（M+H）+	Stearidonic Acid	1.855046538	1.821951222	0.010348702
（M-H）-	Acetylglycine	1.856737087	2.301578311	0.039371677
（M-H）-	Adynerin	3.714841952	0.436638541	0.044124455
（M-H）-	Enterostatin human	3.598108378	0.374013419	0.021397895
（M+H）+	2（1H）-Pyridinone	1.170036687	1.586651424	0.090812831
（M+H）+	4-acetamidobutanoate	1.239515598	3.70826756	0.094628299
（M+H）+	Betaine	4.672929185	1.889313062	0.094288019
（2M+H）+	Chenodeoxycholate	2.756164851	14.32916763	0.070471418

M+	Cholic acid	1.750037262	0.363616361	0.097523359
（M+H）+	Creatinine	4.495529859	1.745276879	0.052984443
（M+H）+	O-Acetyl-L-serine	1.04686378	2.016383749	0.094390408
（M+H）+	Quinaldic acid	1.630836371	0.344681074	0.072419806
（M+H）+	Sphingosine	3.21185588	0.299339595	0.083002248
（M-H）-	11（Z）,14（Z）-Eicosadienoic Acid	2.358367171	0.342700428	0.092090301
（M-H）-	Caprylic acid	4.500113202	2.938060501	0.057905851
（M-H）-	N2-Acetyl-L-ornithine	1.355297847	1.61573553	0.052220441
（M-H）-	Thymidine	7.070189662	0.668402945	0.066306572

Supplementary Table S3. Fecal metabolites with differential abundances between IGR and Control groups

Adduct	Description	VIP	Fold change	*p*-value
（M+H）+	1-Stearoyl-2-hydroxy-sn-glycero-3-phosphoethanolamine	2.522491533	3.049691173	0.023096892
（M+H）+	2（1H）-Pyridinone	1.05511362	1.52860647	0.029200802
（M+H-H2O）+	Phenylacetic acid	2.433085326	0.822987692	0.048946989
（M+H）+	Ser-Glu	1.103930859	0.364108975	0.035450536
（M+H）+	Uracil	1.699052632	0.576042782	0.044324287
（M-H）-	9-OxoODE	3.008546286	2.182475151	0.046825173
（M-H）-	Adynerin	3.90688529	0.324738399	0.026705636
（M-H）-	Cyclohexylsulfamate	4.545393075	1.981069925	0.0426851
（M+H）+	25-hydroxyvitamin D3	2.105398735	1.599025286	0.058959507
（M+H）+	4-Hydroxybutanoic acid 5-lactone	1.570307588	0.74461817	0.096859106
（M+H）+	5,2'-O-dimethylcytidine	1.403353918	0.376896709	0.095726164
（M+H）+	Adenosine 3'-monophosphate	1.492906445	5.348920428	0.064511967
（M+H）+	alpha-Tocopherol	1.719768516	1.883278283	0.079612452
（M+H）+	Arg-Gln	2.644065136	0.483311628	0.068125132
（M+H）+	Bufexamac	2.940144257	2.121800242	0.079173749
（2M+H)+	Chenodeoxycholate	1.446151628	6.787845742	0.090264805

（M+H-H2 O）+	Dihydrotachysterol	1.009083049	1.569077698	0.083516325
（M+H）+	D-Proline	2.643006965	0.566297986	0.096119735
（M+H）+	His-Arg	1.004376093	0.439277992	0.098413794
（M+H）+	His-Lys	1.1243612	0.382235756	0.086039441
（M+H）+	Hypoxanthine	7.868424522	0.542184713	0.07003272
（M+H）+	Ile-Thr	1.625887034	0.381637935	0.053260138
（M+H-H2 O）+	L-Citrulline	1.829002325	0.453004251	0.099173405
（M+H-H2 O）+	L-Proline	1.17176989	0.554228363	0.091841753
（M+H）+	N-Acetyl-L-Histidine	1.568342176	0.41603333	0.092740355
（M+H）+	N-Acetylputrescine	2.076636343	0.207445479	0.080931517
（M+H）+	Pantothenate	3.190989742	0.400749631	0.085065627
（M+CH3 CN+H）+	Pyridoxine	1.12106118	0.787351978	0.053653238
M+	Trp-Arg	1.466918394	0.452682877	0.062637754
（M+H）+	Val-Ala	1.310319439	0.463071583	0.099741118
（M+H）+	Val-Glu	1.682630697	0.479814327	0.070067564
（M+H）+	Val-Ile	7.059090615	0.329551873	0.075154782
（M-H）-	Adenosine 3'-monophosphate	1.41612115	5.039642453	0.055582688
（M-H）-	alpha-hydroxy myristic acid	1.864570317	2.214260128	0.057643216

（M-H）-	D-Alanyl-D-alanine	2.588538897	0.352130608	0.060807648
（M-H）-	gamma-L-Glutamyl-L-valine	6.600047405	0.379523354	0.063359501
（M-H）-	Gentisic acid	2.136191049	0.410504295	0.096461416
（M+Na-2H）-	Inosine	1.390533468	0.445619007	0.087685913
（M-H）-	Isobutyric acid	10.4572771	0.657087992	0.095806054
（M-H）-	L-Alanine	1.316936119	0.501678465	0.065017104
（M+CH3COO）-	Lithocholic acid	2.356699457	3.341101381	0.077969581
（M-H）-	L-Leucine	9.838581558	0.583960081	0.070617027
（M-H）-	L-Valine	5.62725702	0.598233502	0.061971619
（M-H）-	N2-Acetyl-L-ornithine	1.26052294	1.961500507	0.088685418
（M-H）-	Pantothenate	8.462991979	0.523591679	0.08476727
（M-H）-	Phenol	1.002226554	0.425393884	0.062618695
（M-H）-	Propionic acid	5.56365416	0.682137137	0.090270779
（M-H）-	Uracil	6.02378133	0.814747567	0.094342569
（M-H）-	Uridine 5'-monophosphate	2.031721353	7.833531006	0.054713851
（M-H）-	Xanthine	1.695012623	0.631284016	0.057191532

Supplementary Table S4. Enrichment analysis of the differentially abundant pathways between T2DM and Control groups

Map_ID	Map_Name	p.value	FDR	richFactor
mcc01230	Biosynthesis of amino acids	0.0118823	0.126412272	0.0234375
mcc00120	Primary bile acid biosynthesis	0.013556237	0.126412272	0.042553191
mcc04210	Apoptosis	0.015301572	0.126412272	0.25
mcc00061	Fatty acid biosynthesis	0.020225963	0.126412272	0.034482759
mcc00330	Arginine and proline metabolism	0.035141056	0.140642087	0.025641026
mcc04217	Necroptosis	0.037842887	0.140642087	0.1
mcc04122	Sulfur relay system	0.041552409	0.140642087	0.090909091
mcc00785	Lipoic acid metabolism	0.048931263	0.140642087	0.076923077
mcc04976	Bile secretion	0.052228708	0.140642087	0.020618557
mcc04071	Sphingolipid signaling pathway	0.056256835	0.140642087	0.066666667
mcc00220	Arginine biosynthesis	0.085033441	0.176789674	0.043478261
mcc00600	Sphingolipid metabolism	0.092097941	0.176789674	0.04
mcc02010	ABC transporters	0.095635502	0.176789674	0.01459854

Supplementary Table S5. Enrichment analysis of the differentially abundant pathways between IGR and Control groups

Map_ID	Map_Name	p.value	FDR	richFactor
mcc04974	Protein digestion and absorption	4.02424E-08	1.60969E-06	0.14893617
mcc04978	Mineral absorption	5.98838E-05	0.001197675	0.137931034
mcc05230	Central carbon metabolism in cancer	0.00015948	0.002126405	0.108108108
mcc01230	Biosynthesis of amino acids	0.000354198	0.003541975	0.046875
mcc00970	Aminoacyl-tRNA biosynthesis	0.000602889	0.004823109	0.076923077
mcc00770	Pantothenate and CoA biosynthesis	0.001458179	0.009721196	0.1
mcc02010	ABC transporters	0.003540499	0.02023142	0.03649635
mcc00230	Purine metabolism	0.005623142	0.028115709	0.042105263
mcc00290	Valine, leucine and isoleucine biosynthesis	0.013114926	0.052459703	0.086956522
mcc00220	Arginine biosynthesis	0.013114926	0.052459703	0.086956522
mcc00330	Arginine and proline metabolism	0.021289003	0.077414558	0.038461538
mcc00410	beta-Alanine metabolism	0.024631296	0.082104319	0.0625

mcc04150	mTOR signaling pathway	0.030426865	0.093621123	0.25
mcc04977	Vitamin digestion and absorption	0.035594224	0.101697782	0.051282051
mcc00280	Valine, leucine and isoleucine degradation	0.040774491	0.108731977	0.047619048
mcc01100	Metabolic pathways	0.050920008	0.12730002	0.009406657
mcc05152	Tuberculosis	0.067201052	0.158120122	0.111111111
mcc04928	Parathyroid hormone synthesis, secretion and action	0.074391985	0.164547434	0.1
mcc04122	Sulfur relay system	0.081529204	0.164547434	0.090909091
mcc01210	2-Oxocarboxylic acid metabolism	0.082273717	0.164547434	0.02238806
mcc00240	Pyrimidine metabolism	0.088388507	0.168359061	0.030769231
