# Supplementary figures and images for: Alterations in Microbiota and Metabolites Related to Spontaneous Diabetes and Pre-Diabetes in Rhesus Macaques
Source: Genes (Basel). 2022 Aug 24;13(9):1513. doi: 10.3390/genes13091513 (PMC9498908; doi:10.3390/genes13091513)

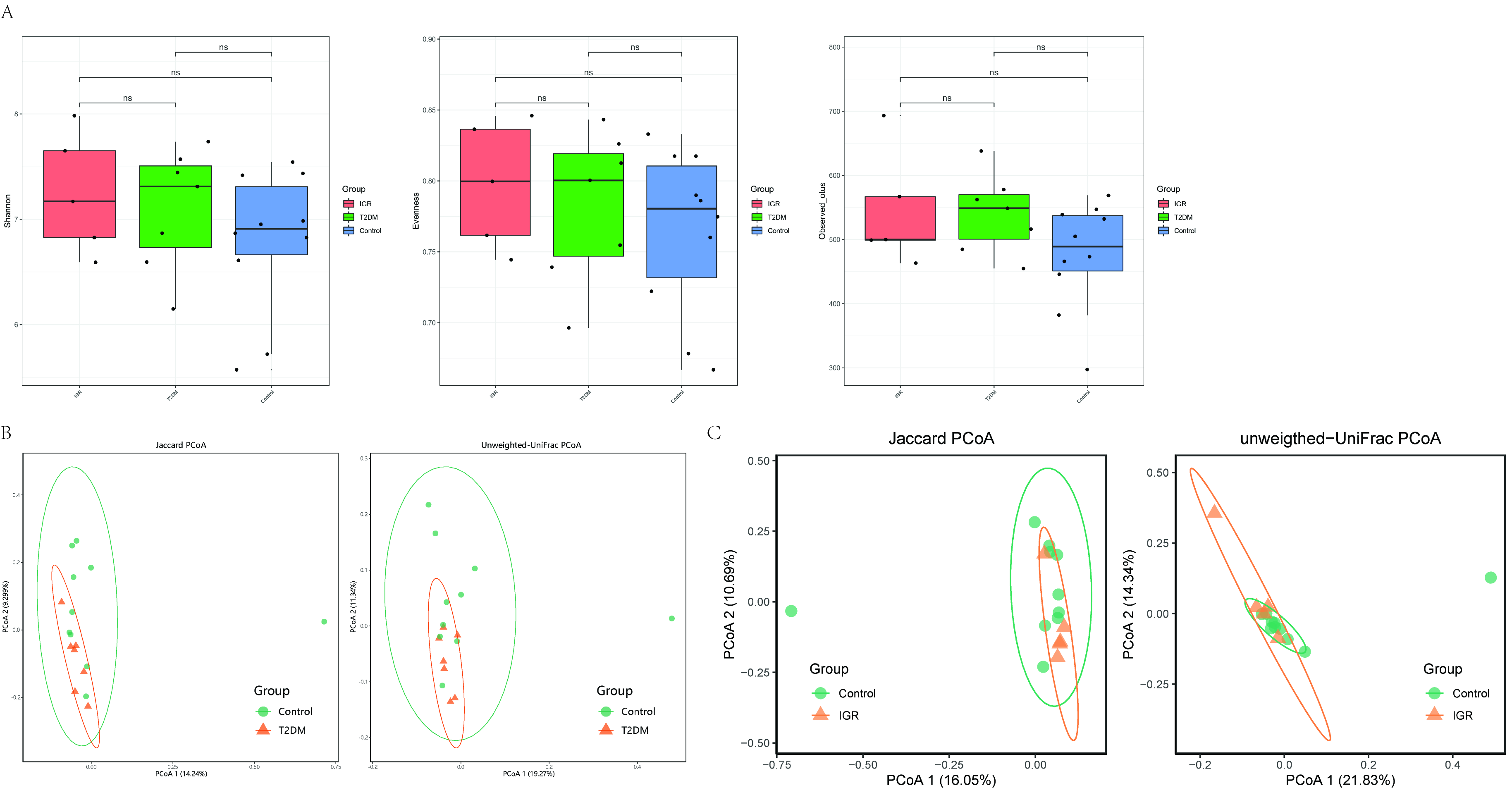

Supplement: Supplementary file 1 [file genes-13-01513-s001.zip › Supplementary Fig. S1.tif]

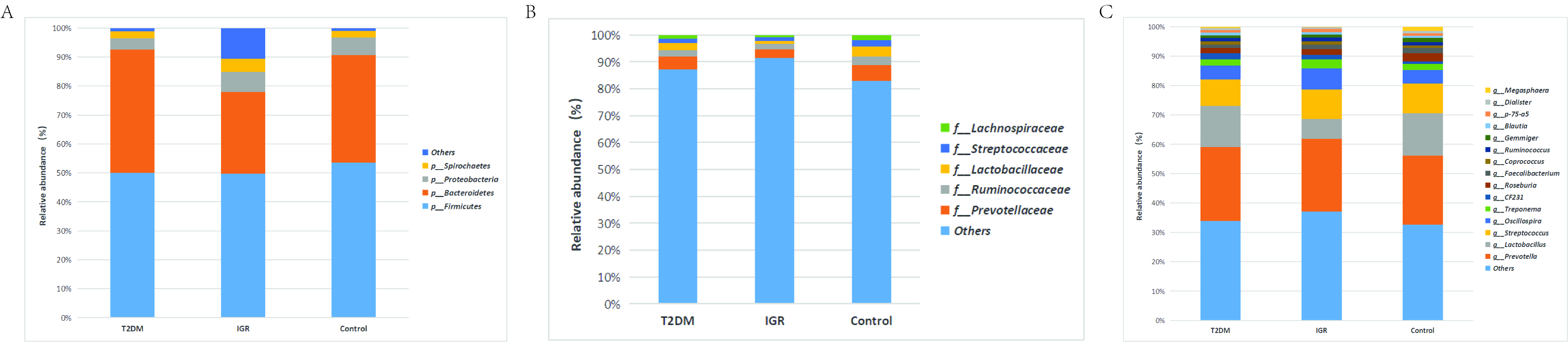

Supplement: Supplementary file 1 [file genes-13-01513-s001.zip › Supplementary Fig. S2.tif]

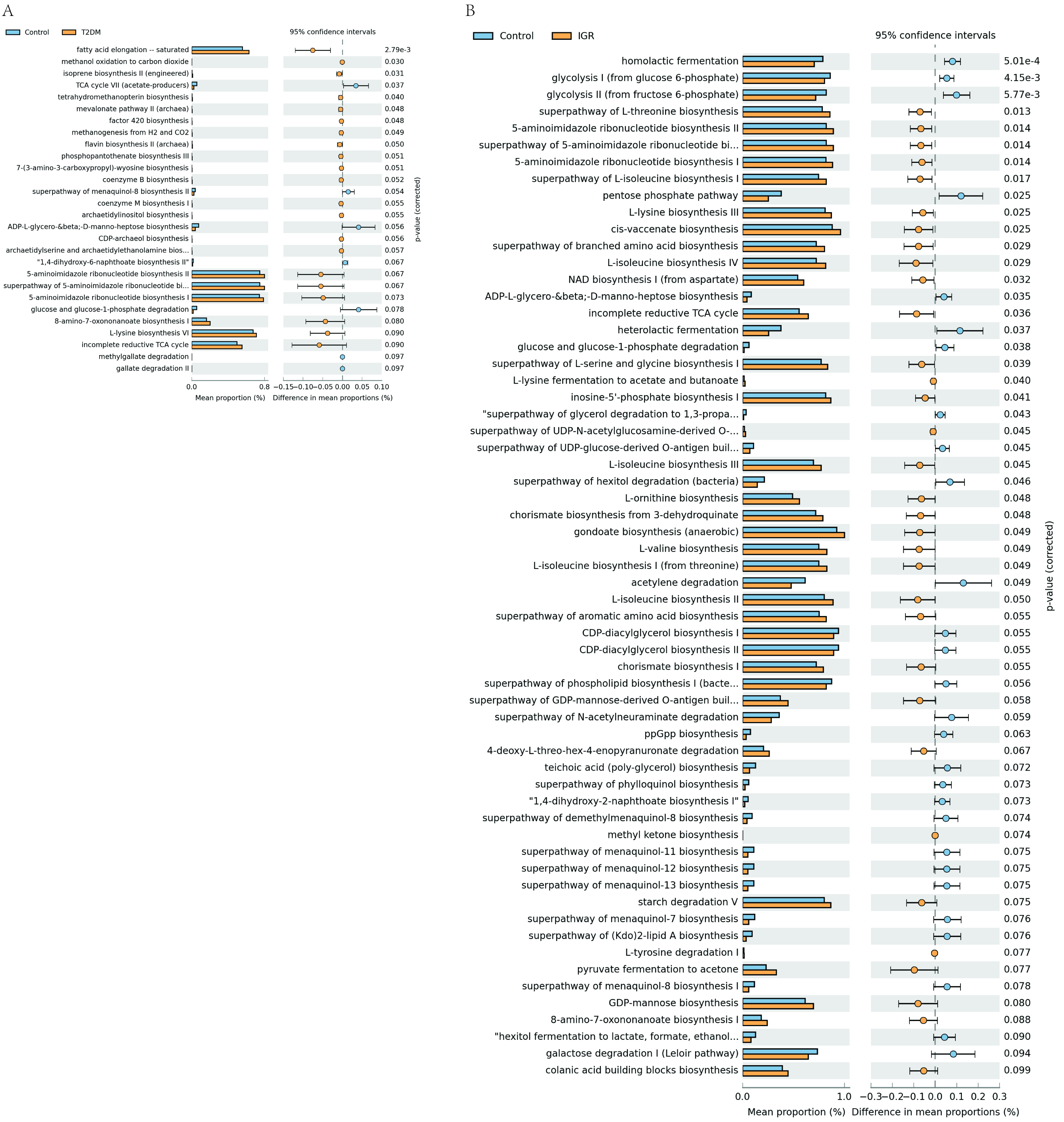

Supplement: Supplementary file 1 [file genes-13-01513-s001.zip › Supplementary Fig. S3.tif]

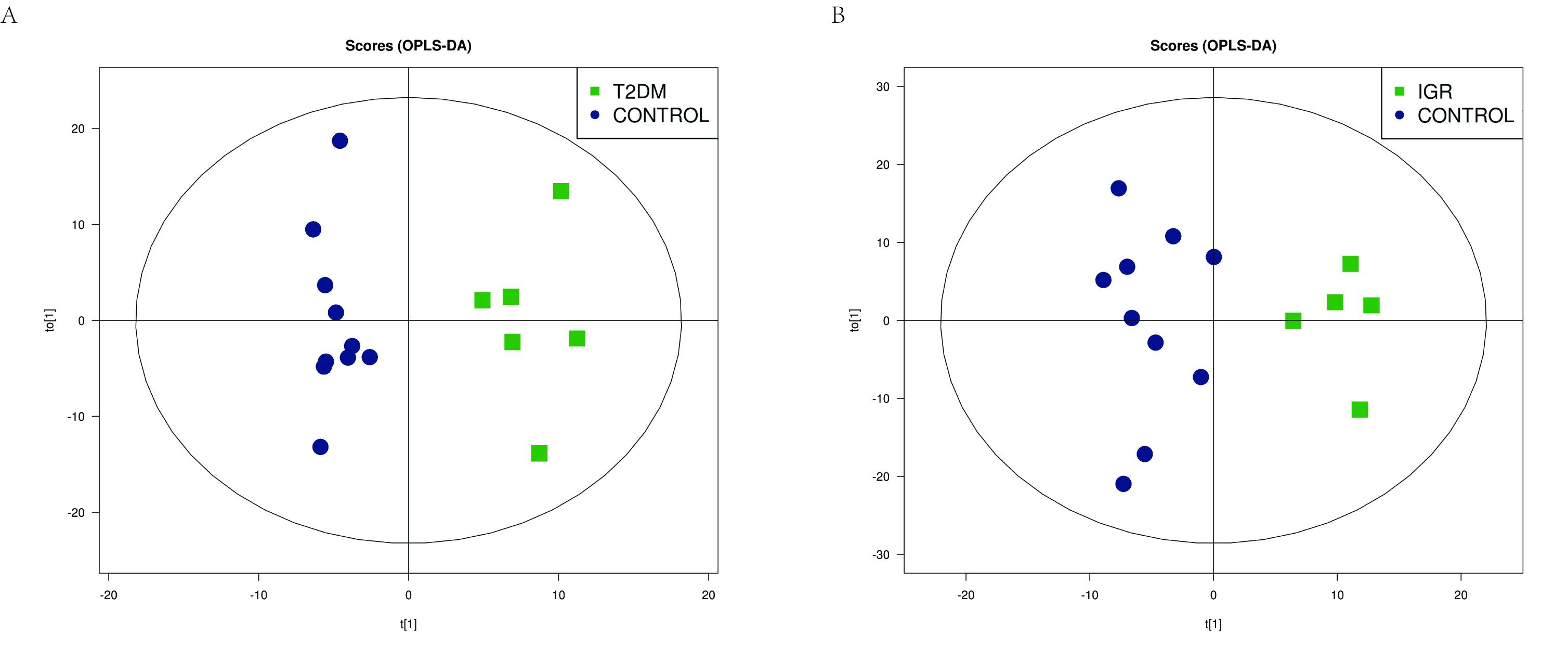

Supplement: Supplementary file 1 [file genes-13-01513-s001.zip › Supplementary Fig. S4.tif]
